# Urban water and electricity demand data for understanding climate change impacts on the water-energy nexus

**DOI:** 10.1038/s41597-024-02930-z

**Published:** 2024-01-23

**Authors:** Renee Obringer, Roshanak Nateghi, Jessica Knee, Kaveh Madani, Rohini Kumar

**Affiliations:** 1 Department of Energy and Mineral Engineering, Pennsylvania State University, University Park, PA 16802 USA; 2 United Nations University Institute for Water, Environment and Health (UNU-INWEH), Hamilton, ON L8P 0A1 Canada; 3 School of Industrial Engineering, Purdue University, West Lafayette, IN 47907 USA; 4 CUNY Remote Sensing Earth Systems (CUNY-CREST) Institute, City College of New York, New York, NY 10031 USA; 5 Department of Computational Hydrosystems, Helmholtz Centre for Environmental Research - UFZ, Leipzig, 04318 Germany

**Keywords:** Climate-change impacts, Civil engineering

## Abstract

As the climate crisis intensifies, it is becoming increasingly important to conduct research aimed at fully understanding the climate change impacts on various infrastructure systems. In particular, the water-electricity demand nexus is a growing area of focus. However, research on the water-electricity demand nexus requires the use of demand data, which can be difficult to obtain, especially across large spatial extents. Here, we present a dataset containing over a decade (2007–2018) of monthly water and electricity consumption data for 46 major US cities (2018 population >250,000). Additionally, we include pre-processed climate data from the North American Regional Reanalysis (NARR) to supplement studies on the relationship between the water-electricity demand nexus and the local climate. This data can be used for a number of studies that require water and/or electricity demand data across long time frames and large spatial extents. The data can also be used to evaluate the possible impacts of climate change on the water-electricity demand nexus by leveraging the relationship between the observed values.

## Background & Summary

Climate change is already causing stress for infrastructure systems, from droughts and heatwaves^1^ to extreme cold snaps^2^. As the crisis continues to intensify, it is critical that researchers work to understand the impacts of climate change on society’s most crucial services. One growing area of research on this topic is the water-energy nexus^3,4^, which has been shown to be significantly impacted by climate^5–8^. Much of the water-energy nexus research has focused on the supply side (e.g., water used for electricity generation), including areas such as hydropower^9,10^ or the energy required to treat and distribute water in urban areas^11^. However, recently, there has been a greater focus on the water-electricity *demand* nexus, which encompasses the many ways in which people consume both water and electricity in their daily lives^12–16^. In terms of climate change adaptation, the existence of the water-energy nexus on both the supply and demand sides means that future plans for utilities must account for the climate change impacts on supply (e.g., will there be enough water for hydropower) *and* the impacts on demand (e.g., will changing precipitation lead to higher consumption of water for landscaping).

Research has shown that extreme events (e.g., heatwaves, droughts, etc.) can shift the demand structures for water and electricity^17–21^, potentially leading to disruptions or outages^22–25^. These disruptions can exacerbate existing vulnerabilities in lower income or marginalized communities, creating public health crises^21,26,27^. Further, there are a number of climate change mitigation and adaption measures, such as electrification and electric vehicle usage, that lead to additional shifts in demand, particularly for the electricity sector. As such, understanding the relationship between the local climate and the water-electricity demand nexus is critical for ensuring the availability of critical services in the face of future climate-induced demand shifts. However, to adequately model this relationship, one requires demand data from utilities, which can be time-consuming and difficult to obtain, particularly at high resolution spatial or time scales.

This challenge is demonstrated in the literature surrounding the urban water-energy nexus, which has often focused on singular cities^7,11^ or small groups of cities^6,15^. This emphasis on small spatial extents does allow for deep analysis on a few cities, but does not allow for larger comparative studies. One of the major reasons for the lack of large-scale studies is the limited availability of data, particularly for water utilities, which are often small, local agencies that do not regularly publish data online. Thus, in order to accurately depict the relationship between the climate and the water-electricity demand nexus across large spatial extents, one would need to contact individual agencies to request data, which may or may not be available. This is a cumbersome task that has led to a significant gap in the water-electricity demand nexus literature. The aim of this study is to close that gap by providing a high quality, large-scale dataset of water and electricity consumption across the United States.

A further challenge arises from the lack of interconnectivity at the utility level, particularly with regard to climate change adaptation plans. In fact, the utility-level interconnections are rarely considered by the separate management agencies, possibly leading to suboptimal decisions^12,28^. This can create issues for climate change adaptation, as the water utilities might not be accounting for changes in the electricity demand, which will in turn impact the water demand. These feedback loops are critical to building resilience to climate change across infrastructure systems, but outside of a few utilities (e.g., Los Angeles Department of Water and Power), many water and electricity systems remain separate. However, by creating a dataset that combines both water and electricity data, we can help close this gap and encourage both researchers and practitioners to leverage the interconnections between water and electricity for better climate change impact assessments and management.

Here, we present a dataset that contains over a decade (2007–2018) of monthly residential water and electricity consumption for 46 major US cities (2018 population >250,000). Additionally, we have included climate data that were obtained from the North American Regional Reanalysis (NARR)^29^ and pre-processed to match the spatial and temporal scale of the consumption data. Finally, we include pre-processed climate data from 57 additional cities in North America that are the most likely climate analogs^30^ of our target cities under RCP2.6 and RCP8.5. This dataset was used in a recent study leverages a climate analog approach to project the water and electricity consumption into the future under climate change^31^. We would like to note that the climate analogs are an example of one means to obtain future climate data estimates for climate change impact assessment, but it is not the only way one could use this data. For example, similar work could be done with general circulation model (GCM) data from the new CMIP6 suite of models. Leveraging the CMIP6 data would require additional effort to download, downscale, and bias-correct the future climate data, which may not be feasible for smaller communities. However, the climate analogs can be found through the original publication^30^, which included 540 North American cities. Additionally, the dataset presented here can be used for any analysis that requires water and/or electricity consumption values at the city-scale, particularly if one is interested in the utility-level interactions between the two demand profiles. In the following sections, we describe the methods used to collect and pre-process the data, the data itself, and a discussion of the validity of the dataset.

## Methods

**Fig. 1:**
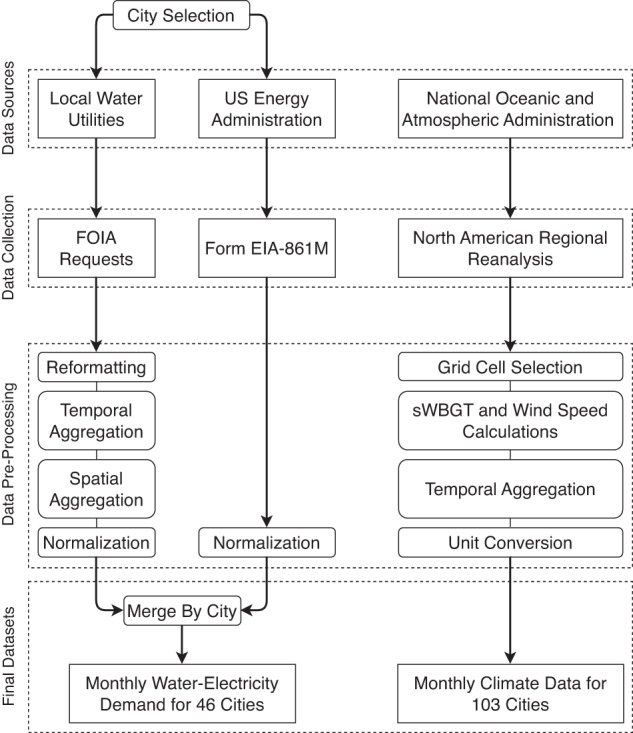
Workflow for collecting and pre-processing the dataset.

This section discusses the data collection process for the curation of this novel dataset. The general workflow is outlined in Fig. 1, as well as discussed in detail in the following sections. Overall, we collected over 10 years of monthly water and electricity consumption data for 46 major US cities. Additionally, we collected and pre-processed climate data for 103 North American cities (46 target cities plus 57 additional analog cities) over the same time period. Most of the data were collected via publicly available sources, but the large spatial extent and differing nature of management in water and electricity systems in the US has prevented the development of large-scale datasets. Here, we present out methods for building one such dataset.

### City selection

**Fig. 2:**
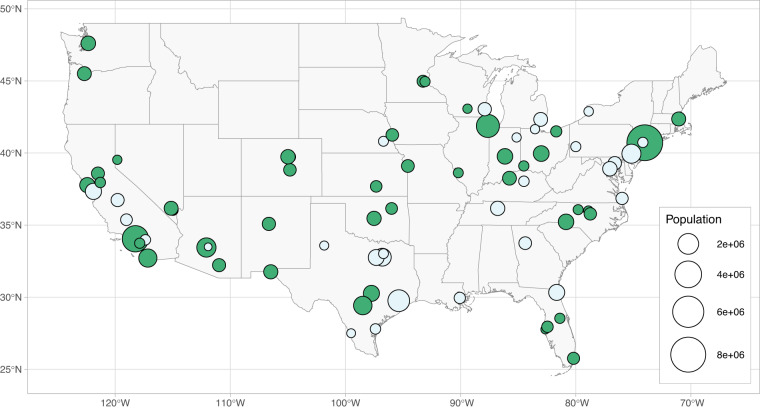
Cities that we initially considered in the data collection phase. The final cities included in the dataset are colored in green. The dots are sized by population.

For curating this dataset, we initially selected 84 cities in the contiguous United States, all of which had a population greater than 250,000 in 2018. The aim was to create a database of the most populous cities in the US, as these cities are often reliant on complex, large-scale water acquisition, treatment, and distribution systems, which may be at risk under climate change. Additionally, the larger cities in the US consume the most electricity, thus can put to significant stress and strain on the larger grid during extreme events. As such, it is critical to obtain data for as many of these cities as possible. Ultimately, we were able to collect data for 46 of these cities, which is discussed below. Figure 2 shows the locations of original cities from which we attempted to collect data as well as the cities in which were successful. Often, the limiting factor on whether a city was included or not was the availability of water consumption data, which we will discuss in detail below.

These cities represent some of the most populous cities in the US, as of 2018; however, in the future, they may not be. Generally, cities in the Southern and Western US are growing rapidly, while other regions are showing more stable populations^32^. Here, we present data for the per capita water and electricity use, but given reliable estimates of population growth (e.g., through shared socioeconomic pathways), one could use the estimated per capita changes to determine possible total changes the utility would experience under climate change.

### Water-electricity consumption data collection & pre-processing

The first step in the work flow was to collect the consumption data. In particular, we collected residential water consumption data and residential electricity consumption data. The process for collecting this data was different for each of the variables. For example, the water consumption data was collected solely through Freedom of Information Act (FOIA) requests, while the electricity consumption data was obtained from the US Energy Administration. Thus, there were more pre-processing steps in the water consumption data than the electricity consumption, which are described below.

#### Water consumption data

**Fig. 3:**
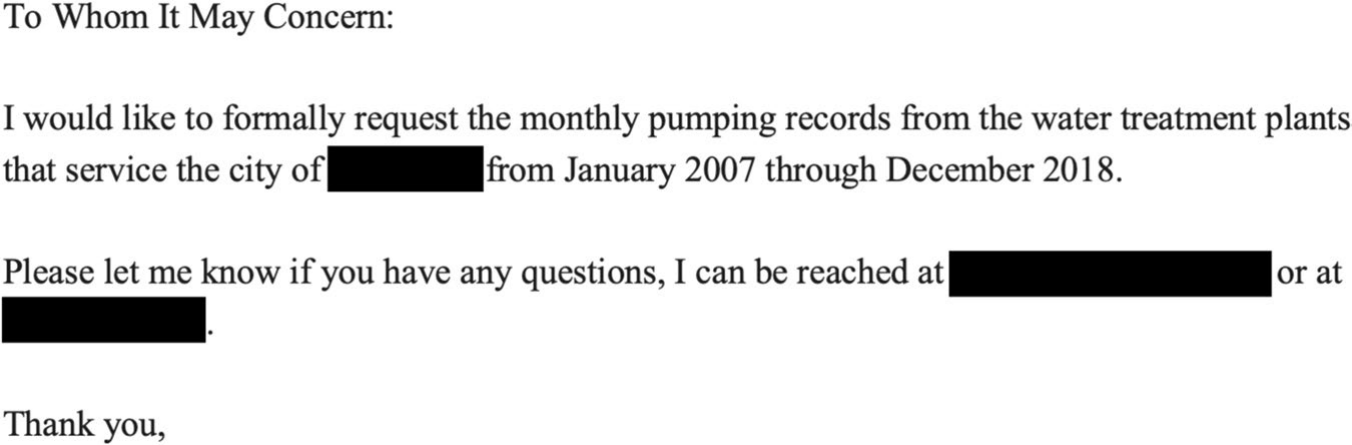
Example of our standard FOIA request sent to cities. Note that the city name and our personal contact information have been removed.

The water consumption data was collected through FOIA requests submitted to local water utilities. The use of FOIA requests as a means to obtain data from water utilities is relatively common, primarily due to the fact that most water utilities are public and there is no national-level database analogous to the EIA databases for electric utilities^33^. In fact, a few recent studies by Chini and Stillwell^33,34^ leverage FOIA data to investigate the electricity used by water and wastewater utilities in the US. This process is, however, quite time consuming and rarely leads to a 100% response rate. In this case, we initially sent requests to 84 major US cities, based on our city selection criteria, discussed above. The text used in the FOIA submission directly requested data from water treatment plant output, which we considered a close proxy for total consumption, though there would be some losses due to leakage in the system that we could not have accounted for. Moreover, we assumed that the majority of the water sold within the city would go towards residential end uses, but the data could have also included commercial, industrial, and wholesale uses, depending on how the city organized their system and pumping records. We did have follow-up conversations with many of the utilities included in this analysis where we discussed our intention of investigating residential demand, however, not all utilities would have been able to separate out residential from other uses. Nonetheless, we normalized the data by the service population, which provides an estimate of how much water is consumed per capita. A sample FOIA request is shown in Fig. 3. We requested monthly data from January 2007 through December 2018. The requests were officially submitted during the first four months of 2019 (January-April).

Ultimately, many of these cities were not included in the final analysis due to lack of data or a lack of response to the request. For example, several cities, such as Washington DC and Henderson (NV), were only able to provide 5 years of data, however, for the analysis we required at least 10 years. Thus, these cities were removed from the analysis. Other cities were removed due to the lack of response. For these cities, we sent up to three follow up emails, after which we removed them from our analysis. In this way, many of the original selected were removed, although some of the cities were removed due to lack of electricity data, which is discussed in the following section.

After collecting the water consumption data, we implemented four pre-processing steps, as shown in Fig. 1. We began with reformatting the data into a spreadsheet form, in the case that the data was provided in a different format. Since the data was obtained through records requests, some cities, such as New York City, sent us copies of their exact records, which were in pdf format. Therefore, the first step was to convert to a spreadsheet, which could be easily read into a computer programming lanugage for analysis. Once all the data was formatted as spreadsheets, we conducted two aggregation steps. The first was temporal aggregation. We wanted the data to be monthly totals, but some cities (e.g., Phoenix, Arizona) provided us daily values that needed to be aggregated to monthly totals. Additionally, in some cities, we needed to spatially aggregate the data from different treatment plants. For example, the City of Columbus (OH) provided us pumping records for each of their three water treatment plants, which we added together to form the city total which is presented here. Finally, the last step of pre-processing was to normalize the dataset by service population. This step was critical for comparing the water and electricity consumption, since different utilities may have different service areas. Thus, we obtained an estimated per capita water consumption, which was used in the analysis.

#### Electricity consumption data

The electricity consumption data was collected through the US Energy Information Administration (EIA). In particular, we used the residential electricity sales data separated by utility, which is collected through Form EIA-861M^35^. In particular, this involved determining the primary utility in a given city and normalizing the monthly residential consumption by the service population. As discussed above, the normalization procedure allowed us to compare the water and electricity consumption on more equal terms. To determine the utilities, we used published maps of service providers to determine which, if any, of the utilities published in the EIA dataset serviced our cities of interest. These utilities are listed in Table 1. While most cities had data published by EIA, some cities did not. Several cities in Texas, for example, did not have electricity consumption data. While EIA collects data from the majority of large utilities, the nature of the Texas electricity market had led to many smaller utilities that are not required to provide data to the EIA. Thus, the cities of Dallas, Fort Worth, and Houston were removed from our analysis due to lack of electricity consumption data. Ultimately, this final cut resulted in 46 cities included in the analysis. Additionally, since the EIA collects, pre-processes, and publishes the data, there were not pre-processing steps needed beyond the normalization procedure. The result was a per capita electricity consumption on a monthly basis between January 2007 and December 2018.

**Table 1:** List of utilities selected for each city based on the published service provider maps.

City of Interest	Utility
Albuquerque, NM	Public Service Company of New Mexico
Aurora, CO	Public Service Company of Colorado (Xcel Energy)
Austin, TX	Austin Energy
Boston, MA	NSTAR Electric (Eversource)
Charlotte, NC	Duke Energy Carolinas
Chicago, IL	Commonwealth Edison
Chula Vista, CA	San Diego Gas & Electric
Cincinnati, OH	Duke Energy Ohio
Cleveland, OH	Cleveland Electric Illuminating Company
Colorado Springs, CO	City of Colorado Springs
Columbus, OH	Ohio Power (AEP)
Denver, CO	Public Service Company of Colorado (Xcel Energy)
Durham, NC	Duke Energy Carolinas
El Paso, TX	EL Paso Electric
Glendale, AZ	Salt River Project
Greensboro, NC	Duke Energy Carolinas
Indianapolis, IN	Indianapolis Power & Light
Kansas City, MO	Kansas City Power & Light (Evergy)
Las Vegas, NV	Nevada Power (NV Energy)
Los Angeles, CA	Los Angeles Department of Water & Power
Lousiville, KY	Louisville Gas and Electric
Madison, WI	Madison Gas & Electric
Miami, FL	Florida Power & Light
Minneapolis, MN	Northern States Power (Xcel Energy)
New York City, NY	Consolidated Edison
Oklahoma City, OK	Oklahoma Gas & Electric
Omaha, NE	Omaha Public Power District
Orlando, FL	Duke Energy Florida
Phoenix, AZ	Salt River Project
Portland, OR	Portland General Electric
Raleigh, NC	Duke Energy Progress
Reno, NV	Sierra Pacific Power (NV Energy)
Sacramento, CA	Sacramento Municipal Utility District
Saint Paul, MN	Northern States Power (Xcel Energy)
San Antonio, TX	City of San Antonio (CPS Energy)
San Diego, CA	San Diego Gas & Electric
San Francisco, CA	Pacific Gas & Electric
Santa Ana, CA	Southern California Edison
Seattle, WA	City of Seattle
St. Louis, MO	Union Electric (Ameren)
Stockton, CA	Pacific Gas & Electric
St. Petersburg, FL	Duke Energy Florida
Tampa, FL	Tampa Electric Company
Tucson, AZ	Tucson Electric Power
Tulsa, OK	Public Service Company of Oklahoma
Wichita, KS	Westar Energy (Evergy)

Parent companies are listed in parentheses.

### Climate data collection & pre-processing

**Fig. 4:**
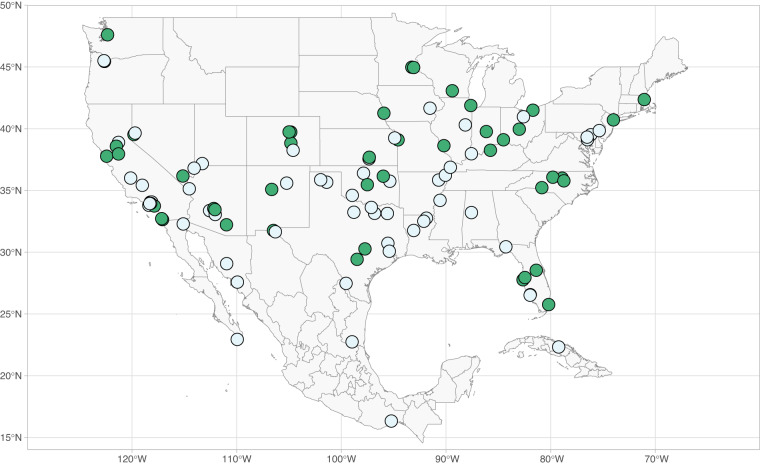
Location of all 103 cities for which we collected climate data. The original 46 cities (i.e., those with water and electricity data) are colored in green.

The climate data were collected from the North American Regional Reanalysis^29^. The steps we took are outlined below. These data were collected for the 46 cities selected above, as well as 57 additional analog cities, shown in Fig. 4. The analogs were selected based on a previous analysis that found the “most likely” climate analog for 540 North American cities^30^. We leveraged these analogs in a separate study, which aimed to use the analogs to project possible changes to the water-electricity demand nexus under climate change^31^.

To extract the NARR data, we first identified the grid cells for each of the 103 cities (46 target plus 57 analogs) in our study. The NARR dataset is available at daily time scale and 0.3° (32 km) spatial resolution. In terms of the climate variables, we collected and processed daily data for dew point temperature, dry bulb temperature, wet bulb globe temperature, relative humidity, precipitation, and the two wind speed components (u and v). The simplified Wet Bulb Globe Temperature (sWBGT) was estimated using information of dry bulb temperature (T) and relative humidity (RH), following Buzan *et al*.^36^ and shown in Eq. 1. Additionally, we calculated the total wind speed from the u and v directional components following Eq. 2.

1 $$sWBGT=0.56T+0.393RH+3.94$$

2 $$speed=\sqrt{{u}^{2}+{v}^{2}}$$

Following the calculations, all selected climatic variables were then aggregated to monthly minimums, means, maximums, and, for precipitation, totals to match the temporal resolution of water and energy datasets. The specific variables and aggregations are outlined in Table 2. Finally, we converted the dry bulb and dew point temperature values from kelvins (K) to degrees Celsius (°C) for consistency across the three temperature variables.

**Table 2:** Input variables and the aggregations considered in this study.

Variable	Units	Aggregations
Dew Point Temperature	K	Minimum, Mean, Maximum
Dry Bulb Temperature	K	Minimum, Mean, Maximum
Wet Bulb Temperature	°C	Minimum, Mean, Maximum
Relative Humidity	%	Minimum, Mean, Maximum
Wind Speed (calculated)	m/s	Minimum, Mean, Maximum
Precipitation	mm/d	Total, Number of Rainy Days

## Data Records

This dataset contains monthly records of per capita water consumption, per capita electricity consumption, and climate data from January 2007 through December 2018 for 46 primary US cities. Additionally, the climate data includes 57 analogs for the primary cities which can be used to estimate changes in the future under high and low warming, without the need for downscaled data from general circulation models (GCMs). The data records, along with code used in a separate analysis, are available via Zenodo^37^.

The data records are separated into two folders: **ClimateData** and **UtilityData**. Within each folder there is one file per city, leading to a total of 149 files (103 climate files plus 46 utility files). All of the data included has been pre-processed following the workflow outlined in Fig. 1. The climate data is available in text files (extension:*.txt*), while the utility data is available in comma-separated values files (extension:*.csv*).

The climate data folder contains 103 text files with pre-processed NARR data. The files follow the naming convention: *city_id_XXX.txt*, with each city getting a unique id number from 001 to 103. We have provided a key for these id values in the **MiscFiles** folder: *cities_loc.txt*. Each file has seven columns, which are described in Table 3. Each column represents a climate variable. Each row represents a day starting from January 1, 2007 through December 31, 2018. The utility data folder contains 46 csv files with pre-processed water and electricity consumption data. The files follow the naming convention: *citynameSTATECODE.csv*, for example, Albuquerque, New Mexico is *albuquerqueNM.csv*. Each file has four columns which are described in Table 4. Each row represents a month from January 2007 through December 2018.

**Table 3:** Climate data attributes.

Column	Description
pre(mm/d)	Daily precipitation in millimeters per day
tavg(K)	Daily average air temperature in kelvin
sWBGT(degC)	Daily average wet bulb temperature in degrees Celsius
dewpoint(K)	Daily average dew point temperature in kelvin
rel_hum(%)	Daily average relative humidity in percent
u-Wind_at_10 m(m/s)	Daily average u-wind speed at 10 m in meters per second
v-Wind_at_10m(m/s)	Daily average v-wind speed at 10m in meters per second

**Table 4:** Utility data attributes.

Column	Description
year	Year in YYYY format
month	Month as a number (1 through 12)
wateruse	Pre-processed monthly water use in gallons per capita
elecuse	Pre-processed monthly electricity use in megawatthours per capita

## Technical Validation

The data collected here was primarily obtained from publicly available sources, some of which conduct their own technical validation. For example, the EIA implements an in-house validation of their electricity sales data (Form EIA-861M), which we used to determine the electricity consumption for each city. Likewise, NARR runs their own validation technique to ensure their reanalysis data is accurate. As such, the primary validation conducted within this dataset was for the water consumption data.

As shown in Fig. 1, after collecting the water consumption data through FOIA requests, we began by reformatting the data into spreadsheets. The majority of the cities provided data in spreadsheet form, but a few provided pdf formatted data. In these cases, we used an export function to export the pdf data into excel, then ran an initial check for transcription errors (e.g., removing text cells, incorrect decimal placement, incorrect columns). We then uploaded the data into RStudio and built a few preliminary plots to visually inspect the data. We looked for outliers, as well as unusual patterns, which we then confirmed were present in the unaltered datasets (i.e., not a transcription error). Finally, we compared the average per capita water consumption with other published values, both in the scientific literature and on government agency websites. Through these steps, we confirmed that the city-level water data we obtained through FOIA requests was accurate and without transcription errors.

## Usage Notes

The water and electricity consumption data are available via csv files online^37^. These files can be opened through spreadsheet software, such as Microsoft Excel, or read into programming interfaces, such as RStudio. The climate data are available via text files online^37^. Similarly, these files can be opened through several applications, including spreadsheets or text editors, as well as uploaded to programming interfaces. For users interested in working with multiple cities, we recommend uploaded the data to the programming interface of your choice (e.g., RStudio, Python IDEs, MATLAB, etc.) as a large data object. An example of this means of upload is available in the R script, *climateanaloganalysis.R*, which has been uploaded alongside the data^37^. In particular, at Line 54 in the R Script, we provide a custom function for reading in a directory of text files into a single list object. A similar piece of code is available at Line 75 for reading in a directory of csv files. For user convenience, we have kept the two types of files in separate folders, so entire directories can be uploaded at once. Additionally, the R script has been set up so that users only need to change the primary file path (i.e., that of the large folder) at Line 37 in order to run the code on their own local machine. The remaining folders and directories have been pre-coded as relative file paths to this main path variable. Finally, we have added README files to each folder within the database, which provide details on the data, as well as any important information for working with the data (e.g., naming conventions, locations of related files, etc.).

### Limitations

The primary purpose of this dataset is to provide a centralized location for water-energy-climate data at the urban level. This data, particularly the water consumption, is not easily available for analysis, which prevents researchers from conducting large-scale analyses related to water-energy systems^33^. This dataset can help alleviate such issues. However, there are some limitations. For example, this dataset only contains urban water and electricity consumption data. There is not any consideration for agricultural or large industrial uses that may also impact the water-energy nexus. Likewise, the water consumption data was requested at the water treatment plant outflow point, with the assumption that all the water that leaves the water treatment plant makes it to households. This assumption does not account for leakage throughout the system, which can be quite large in certain areas of the country. Additionally, although we normalized the data to demonstrate the per capita water consumption across the cities, it does not mean that all the water is being used in residential settings. In particular, the wording of our FOIA request means that many utilities provided the pumping data for all their customers, including commercial, small industrial, and possibly wholesale. That being said, by normalizing by the service population, we were able to investigate the impact of climate change on the average water consumed per capita across the cities, regardless of whether that person is consuming the water at home or in a commercial office space. For a more specific analysis, one would need to request actual billing data, which are often protected under privacy clauses and not available via FOIA requests. Nonetheless, this dataset represents a large collection of monthly water consumption at the city-level across the United States. As such, it is a meaningful addition to the growing body of work in the field that will allow researchers to develop large scale studies on urban water-electricity systems.

In addition to the water data, the electricity data represent the other major piece of data presented in this dataset. While we collected the electricity data from the US Energy Information Administration, a reputable source with internal data checking procedures, there remain uncertainties and limitations with the dataset. First, the EIA collects data from utilities through a survey to a utilities. This survey requires utilities to enter in their monthly data for electricity sales, which could lead to typos or other errors associated with how the data is entered. Further, the EIA states that if exact data are not available, utilities must report the estimated data. This adds a layer of uncertainty, as the estimates could vary greatly, dependent on the process used to generate estimates. Second, not all utilities are sampled each month, a decision aimed at reducing the burden of reporting for the utilities. In these cases, the EIA uses statistical imputation to determine these values for the non-sampled utilities. That being said, most utilities do respond to the annual survey, which the EIA uses to replace imputed monthly values. Finally, although EIA does have stringent data-checking processes for erroneous or missing data, it is not always possible to go back to the utility and correct these issues prior to publication. In these cases, the EIA leverages the same imputation technique for non-respondents to estimate the correct values for that utility. Nonetheless, the EIA goes through a well-documented procedure for checking the data submitted through the surveys, so the data is considered trustworthy. As such, the acquisition and integration of this data with water consumption can be beneficial for future studies interested in the water-electricity nexus.

In this study, we focused on the city-level water and electricity consumption data. To this end, we excluded the larger scale agencies that manage water and electricity across regions. For example, federal and inter-state agencies, such as the Army Corps of Engineers or the Bureau of Reclamation, manage large reservoirs important for inter-state water management. Likewise, the Regional Transmission Organizations (RTOs) and Independent System Operators (ISOs) manage the electric grid at regional scales, controlling electricity transmission across state lines. These larger agencies also have interdependencies, particularly where hydroelectric power is generated, however, it is beyond the scope of this data descriptor to account for these larger interconnections.

Finally, the climate data included in this study was obtained through the use of climate analogs^30^, rather than the traditional GCM-based approach. While the water and electricity use data could be integrated with GCM data, it is beyond the scope of this data descriptor to provide such data. That being said, there are some limitations to the climate analogs approach. First, the climate analogs need to be developed separately. Thus, if a given city does not have a known analog, it could be difficult to determine the analogs themselves. However, Fitzpatrick and Dunn^30^ developed analogs for 540 North American cities, which can greatly improves the usability of these analogs. Another limitation of the analog approach is that analogs are often created for a single point in the future, rather than a time series. Thus, the analogs are able to be used to assess the climate change impacts at a single point (e.g., the year 2080) compared to our current time, but without a time series of analogs, one cannot assess how those impacts may evolve over time. Meanwhile, GCM-derived data does contain a time series of points, so it would be possible to assess changes over time. Nonetheless, most impact assessment tools are focused on the average changes over the course of a single time period, thus the analogs can still be used as a proxy for these common assessments, given the users recognize this caveat.

### Data examples

**Fig. 5:**
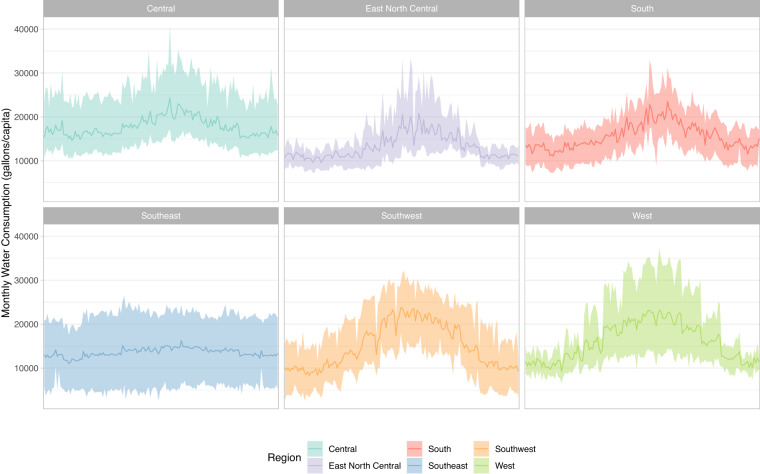
Example of water consumption data, separated by region. The ribbon shows the maximum and minimum across the region, while the darker line shows the mean.

**Fig. 6:**
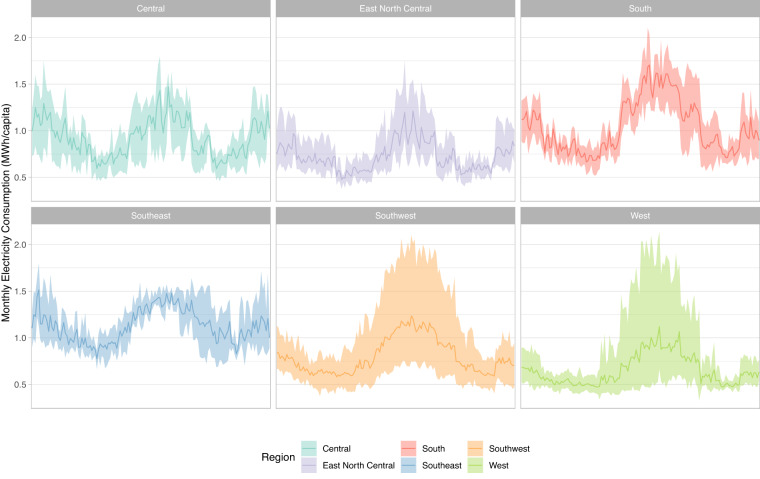
Example of electricity consumption data, separated by region. The ribbon shows the maximum and minimum across the region, while the darker line shows the mean.

Examples of the water and electricity consumption data are shown in Figs. 5, 6, respectively. These figures show the montly consumption values, separated by NOAA climate regions. The ribbons show the full spectrum of consumption throughout all the cities of a given region, while the lines show the mean consumption. We used this data to model the climate-water-energy relationship for urban water and electricity demand. Future work could use this data to better understand a number of water-energy relationships within large US cities.

## Data Availability

The code for pre-processing the data, as well as the subsequent analyses, is available online^37^. The code was written in R version 4.1.2 and last ran on April 16, 2022.
